# *Citrullus colocynthis* Seed Ameliorates Layer Performance and Immune Response under Acute Oxidative Stress Induced by Paraquat Injection

**DOI:** 10.3390/ani12080945

**Published:** 2022-04-07

**Authors:** Ahmed O. Abbas, Abdulaziz A. Alaqil, Nancy N. Kamel, Eman S. Moustafa

**Affiliations:** 1 Department of Animal and Fish Production, College of Agricultural and Food Sciences, King Faisal University, P.O. Box 420, Al-Ahsa 31982, Saudi Arabia; aalaqil@kfu.edu.sa; 2 Department of Animal Production, Faculty of Agriculture, Cairo University, Giza 12613, Egypt; emansm23@agr.cu.edu.eg; 3 Department of Animal Production, National Research Center, El Buhouth St., Dokki, Giza 12622, Egypt

**Keywords:** colocynth, paraquat, acute oxidative stress, immune response, stress biomarker, laying hen

## Abstract

**Simple Summary:**

In recent years, natural, plant-based antioxidants have been increasingly popular among poultry producers to boost production and welfare. Colocynth, i.e., *Citrullus colocynthis*, is an herbaceous plant known to have antioxidant properties. Employing laying hens, this study investigated the potency of dietary colocynth seed supplementation to reduce the deleterious effects of acute oxidative stress induced by paraquat injection. The results demonstrated that supplementing layers’ diets with colocynth seed at 0.1% alleviated oxidative stress responses and significantly improved egg production performance. Furthermore, the immunological responses of the acute-oxidative-stressed layers were enhanced with colocynth seed supplementation. Thus, the inclusion of colocynth seed in layer chickens’ diets can improve egg production performance, restore the redox balance, and enhance immunological responses when they are reared under acute oxidative stress conditions.

**Abstract:**

Oxidative stress is a detrimental physiological state that threatens birds’ productivity and general health. Colocynth is an herbal plant known for its bioactive properties, and it is mainly known for its antioxidant effects. This study’s purpose was to investigate how effective colocynth seed is at lowering the detrimental impact of acute oxidative stress caused by paraquat (PQ) injection in laying hens. A total of 360 Hy-Line Brown chickens, aged 39 weeks, were gathered and divided into four equal groups (10 hens × 9 replicates) in a 2 × 2 factorial design. The experimental groups were given either a basal diet or the basal diet supplemented with colocynth seed (1% of diet). Starting from week 40 of age and for 7 successive days, the experimental groups were either injected daily with paraquat (5 mg/kg body weight) or with saline (0.5 mL, 0.9% NaCl). Egg production performance with selected stress biomarkers and immunological response parameters were investigated at the end of week 40 of age. Our data revealed a significant reduction in egg production with an increase in blood stress biomarkers (i.e., HSP-70, corticosterone, and H/L ratio) in PQ-injected groups compared with non-stressed groups. Furthermore, an unbalanced redox state was detected in acute oxidative stress groups, with a significant rise in lipid peroxidation level, a reduction in total antioxidant capacity (TAC), and a drop in superoxide dismutase (SOD) and catalase enzyme activity. Supplementing PQ-injected hens with colocynth seed reduced the deleterious effects of acute oxidative stress. There was a significant drop in stress biomarkers with a significant rise in antioxidant enzyme activity and TAC observed in the PQ-injected group provided with colocynth seed supplementation. Remarkably, supplementation of colocynth in the non-stressed group resulted in a significant 27% increase in TAC concentration and 17% higher SOD activity when compared with the non-stressed control group. Colocynth supplementation in the PQ-injected group elevated the total white blood cell count by 25% and improved the B-lymphocyte proliferation index (a 1.3-fold increase) compared with the PQ-injected group that did not receive supplementation. Moreover, the non-stressed colocynth-supplemented group had significantly higher cell-mediated and humoral immune responses than the non-stressed control group. This study demonstrated that colocynth seed supplementation in birds exposed to acute oxidative stress may effectively alleviate its negative impacts on production performance, immunological responses, and redox status. We also inferred that, under normal conditions, colocynth seed can be added to laying hens’ diets to stimulate production and ameliorate immune responses.

## 1. Introduction

Oxidative stress exposure is detrimental to poultry immunological responses, redox status, production performance, and well-being [1,2,3]. During the progression of oxidative stress, the excessive production of reactive oxygen/nitrogen species (RONS) causes direct damage to cell macromolecules, such as lipids, proteins, and DNA, and eventually cell death [4,5]. In poultry, oxidative stress has also been linked to immunosuppression, gut health disorders, and a decrease in productive and reproductive functions [6]. Moreover, stress exposure, such as environmental or nutritional stress, has been associated with decreased feed efficiency and weight gain with increased mortality rates [7,8]. On one hand, severe immune abnormalities are associated with oxidative stress induction [2,9]. These abnormalities include the severe suppression of cell- and humoral-mediated immunity and lymphocyte proliferation and a reduction in immunoglobulin levels. On the other hand, redox status is crucial for maintaining proper cell signaling and is implicated in cell stress adaption [6]. Accordingly, irrespective of stress type, several researchers have reported an association between the onset of excessive RONS production and a reduction in total antioxidant capacity and inhibition in endogenous antioxidant enzyme activity (e.g., SOD and catalase), which leads to imbalances in redox status [10,11,12]. These unfavorable effects of oxidative stress impose a huge challenge on poultry breeders trying to sustain optimum high-quality production, especially under intensive production systems.

Paraquat (PQ), a non-selective herbicide, was introduced as an oxidative stress generator in different in vitro [13] and in vivo [9,14,15] experimental studies. PQ toxicity is based on oxidation–reduction cycle generation, which leads to excessive superoxide radical production, triggering inflammation responses and oxidative stress in different body tissues [16]. Mice injected with PQ, owing to its excessive RONS generation, showed activation of the hypothalamic–pituitary–adrenal (HPA) axis, as well as stimulation of nuclear factor kappa B (NF-κB) and heat shock protein 70 (HSP-70) production [17]. Additionally, PQ is involved in the inhibition of nuclear factor 2 (Nrf2, erythroid-derived 2) activity, with a low expression of antioxidant enzyme genes (e.g., SOD and catalase) in PQ-treated human embryonic neural cell cultures [13]. The relation between oxidative stress and inflammation has been reviewed, and Nrf2 (an antioxidant response factor) and NF-κB (a stress response factor) are the key transcription factors involved in the activation of genes responsible for oxidative stress and inflammation [6,18]. Hence, in order to reduce the negative impacts of oxidative stress generated by PQ, an antioxidant and anti-inflammation treatment agent is required.

Recently, natural antioxidant plant-derived feed additives have been intensively explored to combat menacing oxidative stress states [19,20,21,22,23,24,25,26]. Moreover, several reviews indicated the importance of phytochemical, non-nutritive compounds found in plants as natural antioxidants with other potential bioactive properties [18,27]. Colocynth (*Citrullus colocynthis*) is a traditional medicinal herbal plant found in different desert regions worldwide [28]. Several biological activities have been reported for different colocynth plant parts, such as antioxidant, anti-inflammatory, antimicrobial, anticancer, hypolipidemic, and immune-modulating [28,29,30,31,32,33]. However, the inclusion of colocynth seed into poultry feed has scarcely been investigated. Colocynth seed supplementation was proposed as a potential immune modulator for broilers reared under oxidative stress induced by chronic cyclic heat stress [34]. Furthermore, feeding colocynth seed ethanolic extract (up to 0.2% of the diet) to laying hens, reared under normal environmental conditions, was proposed as a potentially useful nutritional strategy for producing low-cholesterol eggs and to alleviate the biological stress induced by intensive commercial egg production [30]. Aside from that, no investigation has been performed to study the bioactive potential of the dietary addition of colocynth seed on laying hens’ production performance and immunological response under extreme oxidative stress conditions. Thus, this study was designed to investigate the potency of dietary colocynth seed supplementation to overcome the negative impacts of oxidative stress induced by PQ injection on production performance, immunological responses, and the retrieval of redox balance.

## 2. Materials and Methods

### 2.1. Ethical Statement

The present experimental design and methodology were reviewed and approved by the Research Ethics Committee (REC) at King Faisal University (KFU-REC-2022-MAR-EA000480). The paraquat dosage was tested to be sublethal dose that generated reactive oxygen species with no dangerous effects on birds’ lives. However, cervical dislocation was applied to allow humane endpoints if any bird showed extreme stress signs.

### 2.2. Experimental Design and Birds’ Management

A number of 360 apparently healthy commercial Hy-Line Brown layers at 39 weeks of age were recruited and divided into four experimental groups (n = 90; 9 replicates × 10 birds) in a 2 × 2 factorial design. The experimental groups were identified according to stress exposure and diet supplementation as: non-stressed group provided with basal diet (non-stressed control group), non-stressed group provided with *Citrullus Colocynthis* seed supplementation (colocynth-seed-supplemented group), oxidative stress group injected with paraquat and provided with basal diet (PQ-injected group), and oxidative stress group injected with paraquat and provided with *C. Colocynthis* seed supplementation (colocynth seed supplementation with PQ injected group). Colocynth seed supplementation was administered at 0.1% of the diet starting from week 39 until the end of week 40 of age. Meanwhile, starting at week 40 of age, paraquat was subcutaneously injected at a dose of 5 mg/kg body weight for 7 successive days. The non-PQ-injected experimental groups were injected with 0.5 mL saline (0.9% NaCl) for the same 7 days. Birds’ general management was performed according to the Hy-Line Brown commercial layers management guide (https://www.hyline.com/literature/brown, accessed on 10 January 2022). During the experimental period, the recruited layers were raised in single cages in closed laying house under controlled environment conditions. The ambient temperature was kept at 22 ± 1 °C with 60% relative humidity and a daily lighting program of 16 L:8 D. Feed and water were freely accessible at all times during the experimental period.

### 2.3. Colocynth Seed Proximate Analysis and Phenolic Compounds and Fatty Acids Profiles

Colocynth seed sample was analyzed to determine the key nutritive compounds following the official methods of analysis [35]. The dry matter (DM), crude protein (CP), ether extract (EE), crude fiber (CF), Ash, and nitrogen-free extract (NFE) of colocynth seed were 93.1, 10.4, 18.5, 49.5, 2.2, and 19.4%, respectively.

The identification of colocynth seed phenolic compounds was performed using high-performance liquid chromatography (HPLC). Sample preparation was carried out according to the method described in [36]. The test method of Agilent Application Note (No. 5991-3801EN; 2014) was conducted using HPLC instrument (Agilent 1260 infinity HPLC Series, Agilent, Palo Alto, CA, USA). The HPLC was equipped with quaternary pump, and the separation was performed with Kinetex^®^ 5 μm EVO C18 100 (100 × 4.6 mm) column (Phenomenex, CA, USA), operated at 30 °C. The injected volume was 20 μL, and the separation was achieved using a ternary linear elution gradient with HPLC-grade water, 0.2% H_3_PO_4_ (*v/v*), methanol, and acetonitrile. The detection of different phenolic compounds was achieved using variable wavelength detector (VWD) set at 284 nm. Colocynth seed phenolic compounds profile identified by HPLC is presented in Table 1.

Colocynth seed oil was extracted and esterified to fatty acid methyl esters (FAME) using the methods described by Kinsella [37]. The colocynth FAME profile was identified using gas chromatography (GC) instrument (GC System HP6890 Series, HP-Hewlett Packard, Palo Alto, CA, USA) under the following conditions: The device was equipped with capillary column (Agilent 19091J-413; HP-5 5% phenyl methyl siloxane) with a 1.5 mL/min initial flow rate under a constant flow mode. The oven initial temperature was programmed at 70 °C with initial time of 1 min. The first ramp reached final temperature of 120 °C at a rate of 40 °C per min. The second ramp reached final temperature of 220 °C at a rate of 8 °C per min. The injection temperature was set at 240 °C. Flame ionization detector (FID) was used at temperature 280 °C using nitrogen as a carrier gas with hydrogen and air flow rate of 30:30:300 mL/min, respectively, under a constant makeup flow mode. Fatty acid methyl esters were differentiated and identified by comparing the relative retention time of sample peaks against a fatty acid methyl ester standard injected under the same conditions. The fatty acids profile of colocynth seed oil identified by GC is presented in Table 2.

### 2.4. Layers Production Performance

Individual hen egg production numbers and weights were recorded for seven successive days starting from week 40 of age. Feed intake was recorded daily by subtracting the residuals from the total offered diet. Egg mass was calculated using the following formula: (Egg mass = egg production number (per week) × average egg weight (per week)), while feed conversion ratio (FCR) was calculated as g feed/g egg mass.

### 2.5. Blood Sampling and Preparation

At the end of week 40, blood samples (n = 9, one bird per group replicate; 4 mL each) were drawn from the wing vein in a heparinized tube. Sufficient amount of each whole blood sample was used to determinate total white blood cell counts (TWBCs) and heterophil to lymphocyte ratios (H/L). To obtain plasma, the rest of blood samples were centrifuged at 4 °C and 1800× *g* for 20 min. Plasma was then collected and kept at −20 °C to determine corticosterone and immunoglobulin levels and blood metabolites.

To isolate peripheral blood mononuclear cells (PBMCs), two sets of freshly drawn blood samples from each experimental group (each set included nine samples, one sample per group replicate; 4 mL each) were collected in a heparinized tube as previously described [9]. The collected PBMC pellets were stored at −80 °C and were used for the determination of HSP-70, redox parameters, and T- and B-lymphocyte proliferation indexes.

### 2.6. White Blood Cell Count and Differentiation

The count of total white blood cells (TWBCs) was performed in whole blood samples (n = 9; one sample per replicate) according to the methods described by Gehad, et al. [38]. Total number of leukocytes was counted by mixing 10 mL of the whole blood with 490 mL of brilliant cresyl blue dye, and then the total number of leukocytes was counted under a microscope at 200× magnification power using a hemocytometer slide.

The heterophil to lymphocyte ratio (H/L) was determined and calculated as described by Mehaisen, et al. [39]. Blood smears (n = 9 per group) were prepared on a clean glass slide and stained with Hema-3 (Fisher Scientific, Pittsburg, PA, USA). The differential leukocyte count for a total of 200 leukocytes was performed in two different slides from each blood sample using a light microscope at a magnification of 100 × with oil immersion. Finally, the heterophil to lymphocyte ratio was calculated.

### 2.7. Redox Status and Stress Biomarkers

The stored PMBC pellets were resuspended in 1 mL of phosphate-buffered saline (PBS) and sonicated to obtain homogenized cells. The supernatants of the homogenized cells were collected after centrifugation at 4 °C and 1030× *g* for 15 min and were used for the following analysis. HSP-70 level was quantified in PMBCs using specific ELISA kit (MBS2702636, MyBioSource, San Diego, CA, USA). The manufacturer’s intra- and interassay coefficients of variation (CV%) were 10% and 12%, respectively, with a detection range of 0.312–20 ng/mL. Malondialdehyde (MDA) levels were determined using quantitative colorimetric assay kits (ab118970; Abcam, Waltham, MA, USA). Superoxide dismutase (SOD) and catalase activity were determined using colorimetric assay kits (ab65354 and ab83464, respectively; Abcam, Waltham, MA, USA). The total antioxidant capacity (TAC) was also determined using colorimetric assay kit (ab65329; Abcam, Waltham, MA, USA).

### 2.8. Plasma Immunoglobulin, Corticosterone, and Blood Metabolite Quantification

According to the manufacturer’s instructions, plasma immunoglobulin (Ig) A, G, and M levels were measured using commercial ELISA kits (Shanghai Jianglai Biotechnology Co., Ltd., Shanghai, China). The assays limits of detection, limits of quantification, intra-assay CV%, and interassay CV% are presented in Table 3 according to the manufacturer-provided data.

Plasma corticosterone level was quantified using chicken-specific quantitative competitive ELISA kit (MBS701668; MyBioSource, San Diego, CA, USA). According to the manufacturer, the CV% for the intra- and interassay were <8% and <10%, respectively, with a detection range of 0.5–20 ng/mL. Meanwhile, plasma total protein (TP), triglyceride (TG), and total cholesterol levels were quantified according to the kits’ protocols using quantitative colorimetric assays (ab102535, ab65336, and ab65390, respectively; Abcam, Waltham, MA, USA). As for liver enzyme activity, alanine amino transferase (ALT) and aspartate amino transferase (AST) activities were determined using commercial kits applying enzyme activity colorimetric assays (ab241035 and ab105135, respectively; Abcam, Waltham, MA, USA).

### 2.9. T- and B-Lymphocyte Proliferation

Stimulating indexes of T- and B-lymphocyte proliferations were determined according to the method described by Mehaisen, Eshak, Elkaiaty, Atta, Mashaly and Abass [39]. The PBMCs were isolated using a separation medium (Histopaque-1077; Sigma-Aldrich, St. Louis, MO, USA). Afterwards, the separated PBMCs were washed and resuspended in complete culture media. The viable lymphocytes were identified and plated at a concentration of 1 × 10^6^ cells per well in 96-well plates. T-lymphocyte proliferation was induced with concanavalin-A (C5275, Sigma-Aldrich, St. Louis, MO, USA), whereas B-lymphocyte proliferation was induced with lipopolysaccharide (L4391, Sigma-Aldrich, St. Louis, MO, USA, USA). Finally, the stimulating indexes (SIs) of T- and B-lymphocyte cells were computed as the optical density of stimulated cells to the optical density of unstimulated control cells.

### 2.10. Statistical Analysis

Data were analyzed using general linear models (GLMs) in SAS 2004 (SAS Institute Inc., Cary, NC, USA). The statistical model included oxidative stress induced by PQ injection, colocynth seed supplementation, and their interaction as fixed effects. To compare means, Duncan’s post hoc test was performed. In order to be considered statistically significant, *p* < 0.05 was set as the significant limit, and the results were expressed as mean ± SEM.

## 3. Results

### 3.1. Egg Production

Egg production parameters indicated that acute oxidative stress exposure negatively affected the layer chickens’ production performance (Table 4). The egg number, weight, and mass of layers subjected to PQ-induced oxidative stress significantly decreased by 29%, 12%, and 38%, respectively, compared with the non-stressed control group. Moreover, feed intake was decreased and the feed conversion ratio (FCR) was impaired with acute oxidative stress exposure. However, the supplementation of colocynth seed in PQ-injected layers showed significant alleviation of its negative impacts on egg production parameters compared with PQ-injected layers on the basal diet, and relative increases in egg number, weight, and mass reached 18%, 7% and 26%, respectively. Interestingly, colocynth seed supplementation to non-stressed layers significantly improved egg production performance compared with the non-stressed control group. Additionally, there was a highly significant interaction between colocynth seed supplementation and PQ injection for egg number, egg mass, and the FCR.

### 3.2. Stress Biomarkers and Redox Status

The physiological stress responses and redox status parameters of layers injected with PQ and supplemented with colocynth seed are presented in Table 5. During the course of acute oxidative stress induction, no severe response was observed in PQ-injected layers. Nevertheless, the acute oxidative stress exposure induced by PQ injection significantly elevated plasma corticosterone concentrations and the H/L ratio with an increase in HSP-70 expression and MDA concentrations, which are all considered as physiological markers of stress. The higher H/L ratio and the elevated concentrations of corticosterone, HSP-70, and MDA in the PQ-injected group were 2.6-, 3.1-, 2.7-, and 2.0-fold, respectively, compared with the non-stressed control group. On the contrary, activities of the antioxidant enzymes SOD and catalase were significantly reduced in the PQ-injected group with a significant decrease in total antioxidant capacity level compared with the non-stressed control group. Altogether, our evidence demonstrated the negative impacts of oxidative stress induced by PQ injection, which consisted of initiating acute stress responses and inducing imbalances in redox status.

However, colocynth seed supplementation to the PQ-injected group significantly alleviated the negative impact of the PQ injection on layers’ stress response and redox status. Colocynth seed supplementation to the PQ-injected group significantly reduced plasma corticosterone concentrations, the H/L ratio, and HSP-70 expression compared with the PQ-injected group that did not receive supplementation. Furthermore, colocynth seed supplementation to the PQ-injected group restored MDA levels and SOD activity, with no significant difference when compared with the non-stressed control group. In addition, colocynth seed supplementation to the non-stressed control group increased TAC concentrations and elevated SOD activity compared with the non-stressed control group. The antioxidant effect of colocynth seed acted independently of oxidative stress exposure, which was indicated by a non-significant interaction between seed supplementation and PQ injection. Nevertheless, the results indicated the positive effects of colocynth seed as a potent natural antioxidant.

### 3.3. Immunological Responses

The acute oxidative stress imposed by PQ injection negatively affected the immunological response of laying hens (Table 6). The PQ-injected group had a significantly lower TWBC count than the non-stressed group. The acute oxidative stress exposure caused by the PQ injection also reduced cell-mediated and humoral immune responses compared with the non-stressed control group. The PQ-injected group had 1.7- and 1.8-fold lower T- and B-lymphocyte proliferation indexes, respectively, than the non-stressed control group. The PQ-injected group also had lower levels of various immunoglobulins than the non-stressed control group, with IgG showing the most significant decline (2.6-fold) followed by IgA (1.7-fold) and IgM (1.5-fold). Conversely, colocynth seed supplementation to the PQ-injected group significantly elevated the TWBC count and improved the B-lymphocyte stimulating index, with no effect on the other measured immunological parameters. Furthermore, colocynth seed supplementation to the non-stressed layer chickens significantly elevated the T- and B-lymphocyte proliferation indexes as well as increased plasma IgA, IgM, and IgG levels compared with the non-stressed control group.

### 3.4. Blood Biochemical Parameters and Liver Enzyme Activity

Table 7 presents blood metabolite and liver enzyme activity values. Results indicated that acute oxidative stress exposure induced by PQ injection did not affect layers’ plasma total protein or triglyceride concentration compared with the non-stressed control group. However, regardless of diet supplementation, the PQ injection significantly increased the concentration of plasma cholesterol by 6.5% in the PQ-injected groups compared with the non-stressed groups. Furthermore, liver ALT and AST enzyme activity significantly increased 1.5- and 1.9-fold, respectively, in the PQ-injected group compared with the non-stressed control group. On the contrary, colocynth seed supplementation with PQ injection significantly reduced liver enzyme activity compared with the PQ-injected group. Irrespective of PQ injection, colocynth seed supplementation significantly reduced blood cholesterol and triglyceride levels compared with the non-supplemented groups, with a reduction percentage ranging from 10% to 21% for cholesterol level and 17% to 22% for triglyceride level.

## 4. Discussion

Modern poultry production is subjected to several stress conditions that subsequently influence productivity and profitability. Oxidative stress generation is mediated by several factors such as unfavorable environmental conditions including heat stress, deficient or contaminated feed, and pathological challenges [40]. Peak egg production is considered a prevalent stressor for commercial layers, and during this period, any extra stress exposure can cause a drop in egg production [8]. A decrease in egg production performance was reported to be associated with oxidative stress exposure induced by *E. coli* injection to laying hens [11]. The reported reduction in egg production was observed during one week after the onset of oxidative stress exposure. Li, et al. [41] stated that laying hens subjected to acute heat stress (35 to 37 °C for 5 days) significantly showed a reduction in laying rates starting from day 3 of heat challenge. Furthermore, they reported a reduction in large yellow and hierarchical follicle numbers with an increase in corticosterone levels and a decrease in estradiol/progesterone ratios in the follicular fluid in small and large yellow follicles [41]. Meanwhile, Wang, et al. [42] noted that an increase in corticosterone levels, in response to stress exposure, suppresses follicular development and decreases the availability of the circulating yolk precursor, which prevents yolk deposition in follicles. The presented results (Table 5) showed a significant 2.7-fold increase in corticosterone concentration in the PQ-injected group compared with the non-stressed control group, which may justify a drop in egg production associated with acute oxidative stress exposure. Thus, the observed drop in egg production number and egg weight in the PQ-injected groups compared with the non-stressed control was justified by the generation of acute oxidative stress state induced by PQ injection [43] and a subsequent increase in corticosterone circulation [42]. Meanwhile, colocynth seed supplementation with PQ injection significantly improved egg production. Furthermore, colocynth supplementation to non-stressed layer chickens significantly improved egg production performance compared with the non-stressed control group. This positive effect of colocynth seed supplementation on egg production performance may be due to its antioxidant properties that alleviate the internal (egg production) and external (PQ injection) oxidative stress impacts on laying hens’ productivity and physiological fitness [30].

Paraquat injection resulted in the induction of severe stress responses, with a significant increase in stress biomarkers (i.e., HSP-70, corticosterone, and H/L ratio). On one hand, Yadawa, Richa and Chaturvedi [17] reported a direct relation between PQ injection and the activation of the hypothalamus–pituitary–adrenal axis with increasing corticosterone circulation levels in PQ-treated mice. They also concluded that the generation of ROS induced by PQ treatment might be responsible for the overexpression of NF-κB and HSP-70 in the mouse hypothalamus. On the other hand, heat shock proteins (HSPs) are molecular chaperones that are progressively produced upon exposure to cytotoxic factors, including xenobiotic and environmental sources, to protect cell proteins from damage [44]. In the present study, PQ exposure imposed acute oxidative stress on the injected layer chickens, which directly caused HSP-70 overexpression. Meanwhile, colocynth seed supplementation to PQ-injected layers reduced the HSP-70 production level due to the alleviation of the oxidative load imposed by PQ injection [45]. Shehata, Saadeldin, Tukur and Habashy [45] concluded that some feed additives have biological properties (e.g., improving oxidative status and anti-inflammatory activity) and can act as HSP gene expression regulators by either the activation or inhibition of their expression after the occurrence of stress.

A reduction in total antioxidant capacity with low antioxidant enzyme activity was associated with PQ injection. Oxidative stress generation may explain the imbalanced redox status associated with PQ injection [10]. By alleviating the oxidative stress imposed by PQ injection, colocynth seed supplementation significantly reduced lipid peroxidation levels as well as improved endogenous antioxidant enzyme activity and increased the total antioxidant capacity. These results may be explained by the previously reported biological antioxidant and anti-inflammation properties of colocynth seed [34,46] and the high amount of phenolic compounds detected by HPLC (Table 1). The scavenging of free radicals is considered an important step in the antioxidant defense approach [47]. Thus, colocynth seed supplementation, owing to its antioxidant bioactivity and high phenolic compound content, was able to scavenge excess ROS induced by PQ injection and retrieve the redox balance [31] as well as increase endogenous antioxidant enzyme activity [34]. The reduction in HSP-70 and the increase in total antioxidant capacity observed in colocynth-seed-supplemented groups imply the activation of the nuclear factor Nrf2 and the inhibition of NF-κB, which elucidates its antioxidant/anti-inflammatory potential [18,48].

Oxidative stress is detrimental to the immune response, causing immunosuppression and severe immune abnormalities [2]. Oxidative stress exposure negatively impacted the immune response of PQ-injected hens. Both cell- and humoral-mediated immune responses were decreased in the PQ-injected groups compared with the non-stressed groups. Paraquat injection was reported to reduce the T-lymphocyte proliferation index and decrease antibody production in turkey poults [9]. Moreover, PQ injection significantly increases the level of a plasma corticosterone that has immunosuppression effects [39]. However, the observed negative impacts of PQ-induced oxidative stress were alleviated with colocynth seed supplementation, which accentuates its immune-modulatory effects previously reported in broilers exposed to oxidative stress induced by heat stress exposure [34]. Additionally, colocynth seed supplementation in non-stressed hens positively enhanced lymphocyte stimulation indexes and immunoglobulin M and G levels, reflecting its immune-stimulating ability for layers reared under standardized conditions.

Blood biochemical compounds are considered indicators of metabolic status. Our data showed no effect of PQ injection on the studied blood biochemical parameters. Meanwhile, colocynth seed supplementation, irrespective of PQ injection, significantly reduced plasma total cholesterol and triglyceride levels. Colocynth seed extract was reported to have a hypolipidemic effect on layers’ plasma and eggs as well as on adipocyte cell cultures [30,49]. Such effects may be justified by the high PUFA content of colocynth seed (65%) identified by our GC analysis. Meanwhile, liver enzyme activity (i.e., AST and ALT) increased in the PQ-injected groups compared with the non-stressed groups. Such an increase reflected the negative impact of PQ injection, which causes liver toxicity and hepatocyte damage. In broiler chickens, Lin, et al. [50] concluded that the liver is an organ that is susceptible to oxidative stress induced by acute heat exposure. Interestingly, colocynth supplementation was able to decrease AST and ALT activities, indicating a potential hepatocyte protective effect.

## 5. Conclusions

This study was the first to investigate the potential of colocynth seed supplementation in alleviating the damaging effects of acute oxidative stress in laying hens. The PQ injection impaired egg production performance, induced acute stress responses, generated redox imbalances, and had immunosuppressive effects. This investigation showed that the antioxidant properties of colocynth seed are able to modulate the negative effects of oxidative stress induced by PQ injection. The positive influences of the antioxidant phenolic compounds and polyunsaturated fatty acid content in colocynth seed reduced the concentrations of oxidative stress markers and elevated antioxidant agents, which resulted in enhanced production performance as well as the retrieval of physiological hemostasis and immune function under oxidative stress. Thus, it was inferred that colocynth seed can be added to layers’ diets at a level of 0.1% to improve egg production performance by alleviating the oxidative stress imposed by intensive production or other internal or external stressors. Considering that this investigation was only a short-term experiment, further studies are recommended to establish solid evidence about the positive effects of colocynth seed as an anti-stressor and to identify the related antioxidant and anti-inflammatory signaling pathways involved in colocynth seed supplementation at the cellular level. 

## Figures and Tables

**Table 1:** Colocynth seed phenolic compounds profile separated and identified by HPLC.

Phenol Name	Concentration (mg/kg)
Pyrogallol	81.524
Gallic acid	210.837
Catechol	330.888
p-Hydroxy benzoic acid	2144.488
Chlorogenic	1436.457
Vanillic acid	322.970
Caffeic acid	635.987
Syringic acid	39.764
p-Coumaric acid	23.214
Benzoic acid	301.348
Ferulic acid	296.652
Rutin	605.829
Ellagic	111.659
o-Coumaric acid	84.237
Quercitin	73.176
Rosmarinic	1135.548
Myricetin	93.736
Kaempherol	5.055

**Table 2:** Fatty acids profile of colocynth seed oil identified and quantified using GC.

Ret. Time	Nomenclature	Fatty Acid %	Fatty Acid Name
8.063	C11:0	0.1320	Decanoatic
9.077	C12:0	2.2775	Lauric acid
9.508	C13:0	0.5606	Triadecylic acid
10.851	C14:0	3.6090	Myristic acid
11.983	C16:0	8.8922	Palmitic acid
15.561	C18:0	9.1458	Stearic acid
16.544	C20:0	0.7511	Arachidic acid
**SFA**		**25.3683**	
10.228	C14:1	3.4284	Myristoleic acid
11.288	C16:1	2.0875	Palmetolic acid
14.453	C18:1	4.2630	Oleic acid
17.504	C20:1	0.1416	Eicosenoic acid
19.571	C22:1	0.1023	Erucic acid
**MUFA**		**10.0229**	
13.048	C18:2n6	56.7404	Trans-Linoleic acid
13.327	C18:2n6	4.0734	Cis-Linoleic acid
17.721	C20:2	0.2396	Eiccosadienoate
18.386	C20:3n6	0.3039	Dihomo-ɣlinolenic acid
19.141	C20:4	0.2459	Arachidonic acid
20.473	C22:2	0.2712	13, 16 Docosadienoic acid
13.751	C18:3	2.6157	Linolenic acid
19.924	C20:5n3	0.1188	Eicosapentaenoic acid
**PUFA**		**64.6088**	

**Table 3:** Immunoglobulin (Ig) assay specifications according to the ELISA kit manufacturer.

Assay Specification	IgA	IgM	IgG
Detection limits, μg/mL	1.0	1.0	1.0
Quantification limits, μg/mL	10	10	25
Intra-assay CV%	10	10	10
Interassay CV%	15	15	15

**Table 4:** Production performance of layers subjected to paraquat-induced oxidative stress (PQ) and dietary *Citrullus colocynthis* seed supplementation (CCs).

Parameter	Non-Stressed (−PQ)		Oxidative Stress (+PQ)		SEM	*p*-Value		
	Basal Diet	CCs	Basal Diet	CCs		CC	PQ	CC × PQ
Egg number, hen/7 days	6.59 ^b^	6.84 ^a^	4.68 ^d^	5.51 ^c^	0.05	<0.0001	<0.0001	<0.0001
Egg weight, g	58.81 ^b^	61.81 ^a^	51.75 ^d^	55.27 ^c^	0.14	<0.0001	<0.0001	0.0645
Feed intake, g/day/hen	113.39 ^b^	116.43 ^a^	107.76 ^d^	110.21 ^c^	0.18	<0.0001	<0.0001	0.1163
Egg mass	387.48 ^b^	422.91 ^a^	242.09 ^d^	304.59 ^c^	2.87	<0.0001	<0.0001	<0.0001
Feed conversion ratio	2.06 ^c^	1.93 ^d^	3.15 ^a^	2.56 ^b^	0.03	<0.0001	<0.0001	<0.0001

PQ, paraquat; CCs, *Citrullus colocynthis* seed; for individual parameters, means with different superscripts significantly differed (*p* < 0.05).

**Table 5:** Stress response of layers subjected to paraquat-induced oxidative stress (PQ) and dietary *Citrullus colocynthis* seed supplementation (CCs).

Parameter	Non-Stressed (−PQ)		Oxidative Stress (+PQ)		SEM	*p*-Value		
	Basal Diet	CCs	Basal Diet	CCs		CC	PQ	CC × PQ
HSP-70, ng/mL	22.15 ^c^	20.67 ^c^	68.51 ^a^	45.25 ^b^	2.22	<0.0001	<0.0001	<0.0001
Corticosterone, pg/mL	5.88 ^c^	4.99 ^c^	16.02 ^a^	10.74 ^b^	0.54	<0.0001	<0.0001	0.0003
H/L ratio	0.32 ^c^	0.31 ^c^	0.82 ^a^	0.64 ^b^	0.03	0.0014	<0.0001	0.0040
MDA, µM/mL	2.59 ^b^	1.89 ^c^	5.21 ^a^	2.95 ^b^	0.18	<0.0001	<0.0001	0.0001
TAC, µM/mL	4.17 ^b^	5.30 ^a^	2.24 ^d^	3.12 ^c^	0.28	0.0011	<0.0001	0.6592
SOD, U/mL	312.22 ^b^	363.89 ^a^	241.00 ^c^	288.11 ^b^	8.48	<0.0001	<0.0001	0.7900
CAT, U/mL	0.83 ^a^	0.92 ^a^	0.61 ^c^	0.73 ^b^	0.03	0.0044	<0.0001	0.6452

PQ, paraquat; CCs, *Citrullus colocynthis* seed; HSP-70, heat shock protein 70; MDA, malondialdehyde; TAC, total antioxidant capacity; SOD, superoxide dismutase; CAT, catalase; for individual parameters, means with different superscripts significantly differed (*p* < 0.05).

**Table 6:** Immunological response of layers subjected to paraquat-induced oxidative stress (PQ) and dietary *Citrullus colocynthis* seed supplementation (CCs).

Parameter	Non-Stressed (−PQ)		Oxidative Stress (+PQ)		SEM	*p*-Value		
	Basal Diet	CCs	Basal Diet	CCs		CC	PQ	CC × PQ
TWBC, ×10^3^/mL	61.46 ^a^	64.25 ^a^	36.40 ^c^	45.64 ^b^	1.83	0.0025	<0.0001	0.0868
SI T-lymphocytes	3.21 ^b^	3.70 ^a^	1.49 ^c^	1.85 ^c^	0.13	0.0024	<0.0001	0.6065
SI B-lymphocytes	2.30 ^b^	2.65 ^a^	1.28 ^d^	1.58 ^c^	0.09	0.0011	<0.0001	0.7668
IgA, μg/mL	170.43 ^a^	179.77 ^a^	98.84 ^b^	109.30 ^b^	10.68	0.3612	<0.0001	0.9584
IgM, μg/mL	449.53 ^b^	554.84 ^a^	301.07 ^c^	338.63 ^c^	21.99	0.0027	<0.0001	0.1333
IgG, mg/mL	1.64 ^b^	2.14 ^a^	0.62 ^c^	0.51 ^c^	0.12	0.1123	<0.0001	0.0171

PQ, paraquat; CCs, *Citrullus colocynthis* seed; for individual parameters, means with different superscripts significantly differed (*p* < 0.05).

**Table 7:** Blood metabolite and liver enzyme activity of layers subjected to paraquat-induced oxidative stress (PQ) and dietary *Citrullus colocynthis* seed supplementation (CCs).

Parameter	Non-Stressed (−PQ)		Oxidative Stress (+PQ)		SEM	*p*-Value		
	Basal Diet	CCs	Basal Diet	CCs		CC	PQ	CC × PQ
Total protein, g/dL	4.51 ^b^	5.22 ^a^	4.97 ^ab^	5.34 ^a^	0.21	0.0122	0.1646	0.4081
Cholesterol, mg/dL	164.44 ^a^	136.00 ^b^	171.56 ^a^	147.44 ^b^	4.05	<0.0001	0.0288	0.5967
Triglycerides, mg/dL	212.56 ^a^	165.56 ^b^	212.11 ^a^	175.11 ^b^	5.46	<0.0001	0.4099	0.3663
ALT, U/mL	10.40 ^c^	9.62 ^c^	15.85 ^a^	13.01 ^b^	0.41	0.0001	<0.0001	0.0184
AST, U/mL	20.09 ^c^	20.04 ^c^	39.03 ^a^	33.66 ^b^	1.16	0.0260	<0.0001	0.0283

PQ, paraquat; CCs, *Citrullus colocynthis* seed; for individual parameters, means with different superscripts significantly differed (*p* < 0.05).

## Data Availability

Not applicable.

## References

[1] Quinteiro W.M., Rodrigues M.V., Ribeiro A., Ferraz-de-Paula V., Pinheiro M.L., Sa L.R.M., Ferreira A.J.P., Palermo-Neto J. (2012). Acute heat stress impairs performance parameters and induces mild intestinal enteritis in broiler chickens: Role of acute hypothalamic-pituitary-adrenal axis activation. J. Anim. Sci..

[2] Hirakawa R., Nurjanah S., Furukawa K., Murai A., Kikusato M., Nochi T., Toyomizu M. (2020). Heat stress causes immune abnormalities via massive damage to effect proliferation and differentiation of lymphocytes in broiler chickens. Front. Vet. Sci..

[3] Roushdy E.M., Zaglool A.W., Hassan F.A.M. (2020). Thermal stress consequences on growth performance, immunological response, antioxidant status, and profitability of finishing broilers: Transcriptomic profile change of stress-related genes. Trop. Anim. Health Prod..

[4] Khansari N., Shakiba Y., Mahmoudi M. (2009). Chronic inflammation and oxidative stress as a major cause of age-related diseases and cancer. Recent Pat. Inflamm. Allergy Drug Discov..

[5] Forcados G.E., Muhammad A., Oladipo O.O., Makama S., Meseko C.A. (2021). Metabolic implications of oxidative stress and inflammatory process in SARS-CoV-2 pathogenesis: Therapeutic potential of natural antioxidants. Front. Cell. Infect. Microbiol..

[6] Surai P.F., Kochish I.I., Kidd M.T. (2021). Redox homeostasis in poultry: Regulatory roles of NF-kappa B. Antioxidants.

[7] Surai P.F., Fisinin V.I. (2016). Vitagenes in poultry production: Part 1. Technological and environmental stresses. Worlds Poult. Sci. J..

[8] Surai P.F., Fisinin V.I. (2016). Vitagenes in poultry production: Part 2. Nutritional and internal stresses. Worlds Poult. Sci. J..

[9] Abass A.O., Kamel N.N., Khalifa W.H., Gouda G.F., El-Manylawi M.A.F., Mehaisen G.M.K., Mashaly M.M. (2017). Propolis supplementation attenuates the negative effects of oxidative stress induced by paraquat injection on productive performance and immune function in turkey poults. Poult. Sci..

[10] Rehman Z.U., Meng C.C., Sun Y.J., Safdar A., Pasha R.H., Munir M., Ding C. (2018). Oxidative stress in poultry: Lessons from the viral infections. Oxidative Med. Cell. Longev..

[11] Abbas A.O., Alaqil A.A., El-Beltagi H.S., Abd El-Atty H.K., Kamel N.N. (2020). Modulating laying hens productivity and immune performance in response to oxidative stress induced by *E. coli* challenge using dietary propolis supplementation. Antioxidants.

[12] Moustafa E.S., Alsanie W.F., Gaber A., Kamel N.N., Alaqil A.A., Abbas A.O. (2021). Blue-green algae (*Spirulina platensis*) alleviates the negative impact of heat stress on broiler production performance and redox status. Animals.

[13] Dou T., Yan M., Wang X., Lu W., Zhao L., Lou D., Wu C., Chang X., Zhou Z. (2016). Nrf2/ARE pathway involved in oxidative stress induced by paraquat in human neural progenitor cells. Oxidative Med. Cell. Longev..

[14] Soni R., Haldar C., Chaturvedi C.M. (2019). Paraquat induced impaired reproductive function and modulation of retinal and extra-retinal photoreceptors in Japanese quail (*Coturnix coturnix japonica*). Comp. Biochem. Physiol. C Toxicol. Pharmacol..

[15] Dostal V., Wood S.D., Thomas C.T., Han Y., Lau E., Lam M.P.Y. (2020). Proteomic signatures of acute oxidative stress response to paraquat in the mouse heart. Sci. Rep..

[16] Tyagi N., Singh R., Chakraborti S., Parinandi N.L., Ghosh R., Ganguly N.K., Chakraborti T. (2020). Paraquat-induced oxidative stress and lung inflammation. Oxidative Stress in Lung Diseases: Volume 2.

[17] Yadawa A.K., Richa R., Chaturvedi C.M. (2019). Herbicide paraquat provokes the stress responses of HPA axis of laboratory mouse, Mus musculus. Pestic. Biochem. Physiol..

[18] Lee M.T., Lin W.C., Lee T.T. (2019). Potential crosstalk of oxidative stress and immune response in poultry through phytochemicals—A review. Asian-Australas. J. Anim. Sci..

[19] Alagawany M., Abd El-Hack M.E., Saeed M., Naveed M., Arain M.A., Arif M., Tiwari R., Khandia R., Khurana S.K., Karthik K. (2020). Nutritional applications and beneficial health applications of green tea and l-theanine in some animal species: A review. J. Anim. Physiol. Anim. Nutr..

[20] Nawab A., Li G.H., An L.L., Wu J., Chao L.W., Xiao M., Zhao Y., Birmani M.W., Ghani M.W. (2019). Effect of curcumin supplementation on TLR4 mediated non-specific immune responses in liver of laying hens under high-temperature conditions. J. Therm. Biol..

[21] Nazar F.N., Videla E.A., Marin R.H. (2019). Thymol supplementation effects on adrenocortical, immune and biochemical variables recovery in Japanese quail after exposure to chronic heat stress. Animal.

[22] Rasouli-Hiq A.A., Bagherzadeh-Kasmani F., Mehri M., Karimi-Torshizi M.A. (2017). *Nigella sativa* (black cumin seed) as a biological detoxifier in diet contaminated with aflatoxin B-1. J. Anim. Physiol. Anim. Nutr..

[23] Ruan D., Fan Q.L., Fouad A.M., Sun Y.Y., Huang S.S., Wu A.J., Lin C.X., Kuang Z.X., Zhang C., Jiang S.Q. (2021). Effects of dietary oregano essential oil supplementation on growth performance, intestinal antioxidative capacity, immunity, and intestinal microbiota in yellow-feathered chickens. J. Anim. Sci..

[24] Abdel-Moneim A.M.E., Shehata A.M., Khidr R.E., Paswan V.K., Ibrahim N.S., El-Ghoul A.A., Aldhumri S.A., Gabr S.A., Mesalam N.M., Elbaz A.M. (2021). Nutritional manipulation to combat heat stress in poultry-A comprehensive review. J. Therm. Biol..

[25] Gessner D.K., Ringseis R., Eder K. (2017). Potential of plant polyphenols to combat oxidative stress and inflammatory processes in farm animals. J. Anim. Physiol. Anim. Nutr..

[26] Huang C.M., Lee T.T. (2018). Immunomodulatory effects of phytogenics in chickens and pigs—A review. Asian-Australas. J. Anim. Sci..

[27] Lee M.T., Lin W.C., Yu B., Lee T.T. (2017). Antioxidant capacity of phytochemicals and their potential effects on oxidative status in animals—A review. Asian-Australas. J. Anim. Sci..

[28] Al-Hwaiti M.S., Alsbou E.M., Abu Sheikha G., Bakchiche B., Pham T.H., Thomas R.H., Bardaweel S.K. (2021). Evaluation of the anticancer activity and fatty acids composition of “Handal” (*Citrullus colocynthis* L.) seed oil, a desert plant from south Jordan. Food Sci. Nutr..

[29] Li Q.-Y., Munawar M., Saeed M., Shen J.-Q., Khan M.S., Noreen S., Alagawany M., Naveed M., Madni A., Li C.-X. (2022). *Citrullus colocynthis* (L.) Schrad (Bitter Apple Fruit): Promising traditional uses, pharmacological effects, aspects, and potential applications. Front. Pharmacol..

[30] Alzarah M.I., Alaqil A.A., Abbas A.O., Nassar F.S., Mehaisen G.M.K., Gouda G.F., Abd El-Atty H.K., Moustafa E.S. (2021). Inclusion of *Citrullus colocynthis* Seed Extract into Diets Induced a Hypolipidemic Effect and Improved Layer Performance. Agriculture.

[31] Bourhia M., Messaoudi M., Bakrim H., Mothana R.A., Sddiqui N.A., Almarfadi O.M., El Mzibri M., Gmouh S., Laglaoui A., Benbacer L. (2020). *Citrullus colocynthis* (L.) Schrad: Chemical characterization, scavenging and cytotoxic activities. Open Chem..

[32] Rizvi T.S., Khan A.L., Ali L., Al-Mawali N., Mabood F., Hussain J., Adnan M., Al-Harrasi A. (2018). In vitro oxidative stress regulatory potential of Citrullus colocynthis and Tephrosia apollinea. Acta Pharm..

[33] Gupta S.C., Tripathi T., Paswan S.K., Agarwal A.G., Rao C.V., Sidhu O.P. (2018). Phytochemical investigation, antioxidant and wound healing activities of *Citrullus colocynthis* (bitter apple). Asian Pac. J. Trop. Biomed..

[34] Alzarah M.I., Althobiati F., Abbas A.O., Mehaisen G.M.K., Kamel N.N. (2021). *Citrullus colocynthis* Seeds: A potential natural immune modulator source for broiler reared under chronic heat stress. Animals.

[35] AOAC (2012). Official Methods of Analysis of AOAC International.

[36] Corrales M., Han J.H., Tauscher B. (2009). Antimicrobial properties of grape seed extracts and their effectiveness after incorporation into pea starch films. Int. J. Food Sci. Technol..

[37] Kinsella J.E. (1966). Metabolic patterns of the fatty acids of *Periplanteta americana* (L.) During its embryonic development. Can. J. Biochem..

[38] Gehad A.E., Mehaisen G.M., Abbas A.O., Mashaly M.M. (2008). The role of light program and melatonin on alleviation of Inflammation induced by lipopolysaccharide injection in broiler chickens. Int. J. Poult. Sci..

[39] Mehaisen G.M., Eshak M.G., Elkaiaty A.M., Atta A.M., Mashaly M.M., Abass A.O. (2017). Comprehensive growth performance, immune function, plasma biochemistry, gene expressions and cell death morphology responses to a daily corticosterone injection course in broiler chickens. PLoS ONE.

[40] Panda A.K., Cherian G. (2014). Role of vitamin E in counteracting oxidative stress in poultry. J. Poult. Sci..

[41] Li G.-M., Liu L.-P., Yin B., Liu Y.-Y., Dong W.-W., Gong S., Zhang J., Tan J.-H. (2020). Heat stress decreases egg production of laying hens by inducing apoptosis of follicular cells via activating the FasL/Fas and TNF-α systems. Poult. Sci..

[42] Wang X.J., Liu L., Zhao J.P., Jiao H.C., Lin H. (2017). Stress impairs the reproduction of laying hens: An involvement of energy. Worlds Poult. Sci. J..

[43] Chen J., Su Y., Lin F., Iqbal M., Mehmood K., Zhang H., Shi D. (2021). Effect of paraquat on cytotoxicity involved in oxidative stress and inflammatory reaction: A review of mechanisms and ecological implications. Ecotoxicol. Environ. Saf..

[44] Kurashova N.A., Madaeva I.M., Kolesnikova L.I. (2020). Expression of HSP70 heat-shock proteins under oxidative stress. Adv. Gerontol..

[45] Shehata A.M., Saadeldin I.M., Tukur H.A., Habashy W.S. (2020). Modulation of heat-shock proteins mediates chicken cell survival against thermal stress. Animals.

[46] Bourhia M., Bouothmany K., Bakrim H., Hadrach S., Salamatullah A.M., Alzahrani A., Alyahya H.K., Albadr N.A., Gmouh S., Laglaoui A. (2021). Chemical Profiling, Antioxidant, Antiproliferative, and Antibacterial Potentials of Chemically Characterized Extract of *Citrullus colocynthis* L. Seeds. Separations.

[47] Surai P.F., Kochish I.I., Fisinin V.I., Kidd M.T. (2019). Antioxidant defence systems and oxidative stress in poultry biology: An update. Antioxidants.

[48] Abo-Al-Ela H.G., El-Kassas S., El-Naggar K., Abdo S.E., Jahejo A.R., Al Wakeel R.A. (2021). Stress and immunity in poultry: Light management and nanotechnology as effective immune enhancers to fight stress. Cell Stress Chaperones.

[49] Jemai R., Drira R., Makni M., Fetoui H., Sakamoto K. (2020). Colocynth (*Citrullus colocynthis*) seed extracts attenuate adipogenesis by down-regulating PPAR gamma/SREBP-1c and C/EBP alpha in 3T3-L1 cells. Food Biosci..

[50] Lin H., Decuypere E., Buyse J. (2006). Acute heat stress induces oxidative stress in broiler chickens. Comp. Biochem. Physiol. Part A Mol. Integr. Physiol..

